# Distinct In Vitro Binding Profile of the Somatostatin Receptor Subtype 2 Antagonist [^177^Lu]Lu-OPS201 Compared to the Agonist [^177^Lu]Lu-DOTA-TATE

**DOI:** 10.3390/ph14121265

**Published:** 2021-12-04

**Authors:** Rosalba Mansi, Pascale Plas, Georges Vauquelin, Melpomeni Fani

**Affiliations:** 1 Division of Radiopharmaceutical Chemistry, Clinic of Radiology and Nuclear Medicine, University Hospital Basel, University of Basel, 4031 Basel, Switzerland; rosalba.mansi@usb.ch; 2 Ipsen Innovation, Translational and Biomarkers Pharmacology, 91140 Les Ulis, France; pascale.plas@ipsen.com; 3 Department of Molecular and Biochemical Pharmacology, Vrije Universiteit Brussel, 1050 Brussels, Belgium; gvauquel@vub.be

**Keywords:** somatostatin receptor, [^177^Lu]Lu-OPS201, [^177^Lu]Lu-DOTA-TATE, radioligand binding, kinetics, binding sites, dissociation

## Abstract

Treatment of neuroendocrine tumours with the radiolabelled somatostatin receptor subtype 2 (SST_2_) peptide agonist [^177^Lu]Lu-DOTA-TATE is effective and well-established. Recent studies suggest improved therapeutic efficacy using the SST_2_ peptide antagonist [^177^Lu]Lu-OPS201. However, little is known about the cellular mechanisms that lead to the observed differences. In the present in vitro study, we compared kinetic binding, saturation binding, competition binding, cellular uptake and release of [^177^Lu]Lu-OPS201 versus [^177^Lu]Lu-DOTA-TATE using HEK cells stably transfected with the human SST_2_. While [^177^Lu]Lu-OPS201 and [^177^Lu]Lu-DOTA-TATE exhibited comparable affinity (K_D_, 0.15 ± 0.003 and 0.08 ± 0.02 nM, respectively), [^177^Lu]Lu-OPS201 recognized four times more binding sites than [^177^Lu]Lu-DOTA-TATE. Competition assays demonstrated that a high concentration of the agonist displaced only 30% of [^177^Lu]Lu-OPS201 bound to HEK-SST_2_ cell membranes; an indication that the antagonist binds to additional sites that are not recognized by the agonist. [^177^Lu]Lu-OPS201 showed faster association and slower dissociation than [^177^Lu]Lu-DOTA-TATE. Whereas most of [^177^Lu]Lu-OPS201 remained at the cell surface, [^177^Lu]Lu-DOTA-TATE was almost completely internalised inside the cell. The present data identified distinct differences between [^177^Lu]Lu-OPS201 and [^177^Lu]Lu-DOTA-TATE regarding the recognition of receptor binding sites (higher for [^177^Lu]Lu-OPS201) and their kinetics (faster association and slower dissociation of [^177^Lu]Lu-OPS201) that explain, to a great extent, the improved therapeutic efficacy of [^177^Lu]Lu-OPS201 compared to [^177^Lu]Lu-DOTA-TATE.

## 1. Introduction

♂ Somatostatin receptors, especially subtype 2 (SST_2_ according to the current nomenclature [1], formerly abbreviated as SSTR2 or SSTR_2_ or sstr_2_), are expressed in high incidence and density in neuroendocrine tumour cells. This serves as the basis of more than 25 years of successful imaging and therapy of neuroendocrine neoplasias (NEN) with radiolabelled peptide analogues of the natural hormone somatostatin. Treatment with radiolabelled SST_2_ agonists, such as [^177^Lu]Lu-DOTA-TATE (^177^Lu-oxodotreotide or Lutathera^®^), where TATE is [Tyr^3^, Thr^8^]-octreotide and DOTA is 1,4,7,10-tetraazacyclododecane-1,4,7,10-tetraacetic acid, is nowadays part of the standard of care of neuroendocrine neoplasms (NENs) [2,3]. However, a number of preclinical and early clinical studies suggest that a significant improvement in both diagnostic performance and therapeutic efficacy can be obtained by the alternative use of radiolabelled SST_2_ antagonists [4,5,6,7].

Indeed, in vivo data have shown that SST_2_ antagonists are associated with higher tumour uptake and tumour radiation dose than SST_2_ agonists, with comparable affinity [5,8]. Among the most studied SST_2_ antagonists is [^177^Lu]Lu-OPS201, also denoted as ^177^Lu-satoreotide tetraxetan or [^177^Lu]Lu-DOTA-JR11 (where JR11 is 4-Cl-Phe-cyclo[dCys-Aph(Hor)-dAph(Cbm)-Lys-Thr-Cys]-dTyr-NH_2_), which was associated with a significantly higher and longer tumour accumulation in vivo as well as a higher DNA double strand breakage in SST_2_-expressing tumours when compared to the agonist [^177^Lu]Lu-DOTA-TATE [5,7,9,10].

Despite these promising in vivo results, little is known still about the molecular and cellular mechanisms that lead to the observed differences between [^177^Lu]Lu-OPS201 and [^177^Lu]Lu-DOTA-TATE, as well as between SST_2_ antagonists and agonists in general. To clarify those differences, the present in vitro study compared the receptor binding characteristics and the cellular processing of [^177^Lu]Lu-OPS201 versus [^177^Lu]Lu-DOTA-TATE in human embryonic kidney (HEK)-293 cells stably transfected with human SST_2_, and their membranes.

## 2. Results

### 2.1. Association Kinetics and Saturation Binding on HEK-SST_2_ Cell Membranes

**Figure 1:**
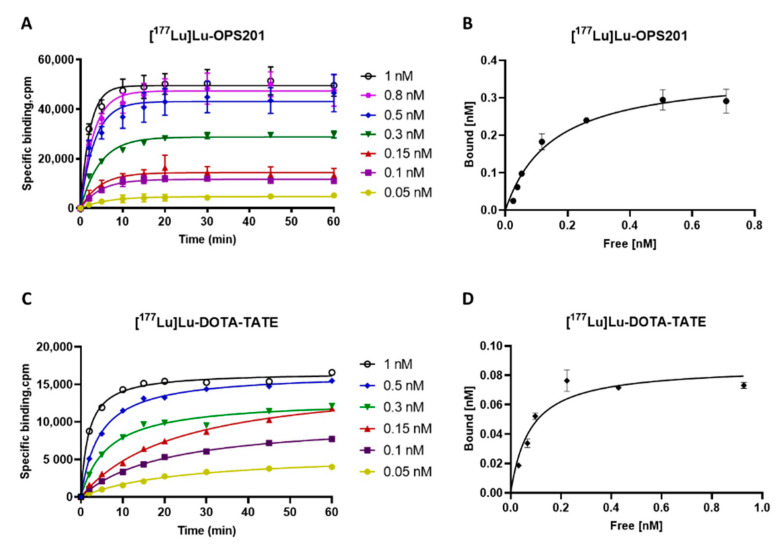
Association versus time plots for specific binding of [^177^Lu]Lu-OPS201 (**A**) and [^177^Lu]Lu-DOTA-TATE (**C**) to HEK-SST_2_ cell membranes (10 µg/well). Membranes were incubated with the indicated concentrations of each radioligand for up to 60 min. Data were fitted using the one-phase exponential association equation. Saturation binding profile for [^177^Lu]Lu-OPS201 (**B**) and [^177^Lu]Lu-DOTA-TATE (**D**). Presented binding values refer to the average of the 20–60 min “equilibrium data” for each radioligand concentration.

The specific, SST_2_ binding of the labelled antagonist [^177^Lu]Lu-OPS201 proceeded swiftly at 37 °C, with equilibrium being reached between 10 min for the highest concentrations and 20 min for the lowest concentrations (Figure 1A). In comparison, the specific binding of the labelled agonist [^177^Lu]Lu-DOTA-TATE was slower, with equilibrium being reached between 20 and 60 min (Figure 1C).

Saturation binding of both radioligands (Figure 1B,D) suited a single-site model, with [^177^Lu]Lu-OPS201 recognising about four times more sites than [^177^Lu]Lu-DOTA-TATE (B_max_ of 0.37 ± 0.02 versus 0.09 ± 0.001 nM, respectively). On the other hand, both radioligands showed comparable affinity (K_D_ of 0.15 ± 0.003 versus 0.08 ± 0.02 nM, respectively).

### 2.2. Dissociation Kinetics on Cell Membranes

**Figure 2:**
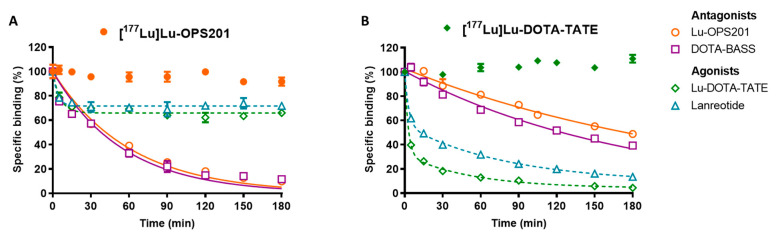
Dissociation of [^177^Lu]Lu-OPS201 (**A**) and [^177^Lu]Lu-DOTA-TATE (**B**) from HEK-SST_2_ cell membranes in the presence of an excess of the unlabelled competitors: antagonists Lu-OPS201 and DOTA-BASS (data points connected with solid lines) and agonists Lu-DOTA-TATE and lanreotide (data points connected with dashed lines). Data are representative of two independent experiments, each performed in triplicate. To control the stability of the radioligand–receptor complexes, membranes were incubated with [^177^Lu]Lu-OPS201 (●) and [^177^Lu]Lu-DOTA-TATE (♦), without the addition of any competitor (indicated by the non-connected solid symbols in both graphs) during the same extended timespan.

The dissociation of [^177^Lu]Lu-OPS201 and [^177^Lu]Lu-DOTA-TATE from HEK-SST_2_ membranes by addition of an excess of unlabelled competitors, the antagonists Lu-OPS201 and DOTA-BASS (DOTA-pNO_2_-Phe-c(dCys-Tyr-dTrp-Lys-Thr-Cys) -dTyr-NH_2_) and the agonists Lu-DOTA-TATE and lanreotide H-d2Nal-c(Cys-Tyr-dTrp-Lys-Val-Cys)-Thr-NH_2_) is shown in Figure 2A,B, respectively.

Specifically bound [^177^Lu]Lu-OPS201 dissociated completely in the presence of Lu-OPS201 following a one-phase exponential decay model (orange solid line in Figure 2A), yielding a t_1/2_ of 41 min. The outcome was very similar in the presence of the alternative antagonist DOTA-BASS (t_1/2_ of 38 min, purple solid line in Figure 2A). By contrast, no more than 30% of this binding could be displaced by a high concentration of the unlabelled agonist Lu-DOTA-TATE (green dashed line in Figure 2A) or of the alternative agonist lanreotide (blue dashed line in Figure 2A). On the other hand, the agonist [^177^Lu]Lu-DOTA-TATE dissociated completely in the presence of its unlabelled counterpart (green dashed line in Figure 2B). However, this dissociation was overtly biphasic, and a two-phase exponential decay model showed that most of the binding dissociated very fast with a t_1/2_ of 1.8 min, while the remainder dissociated with a t_1/2_ of 36 min. The dissociation was also biphasic in the presence of lanreotide with t_1/2_ of 6.2 min for the fast component and 73 min for the slow component (dashed blue line in Figure 2B). Finally, [^177^Lu]Lu-DOTA-TATE dissociated only slowly in the presence of Lu-OPS201 with a t_1/2_ of 180 min (orange solid line in Figure 2B) and in the presence of DOTA-BASS (t_1/2_ of 120 min, purple solid line in Figure 2B). The dissociation parameters are summarised in Table 1.

### 2.3. Cellular Uptake and Internalisation

**Figure 3:**
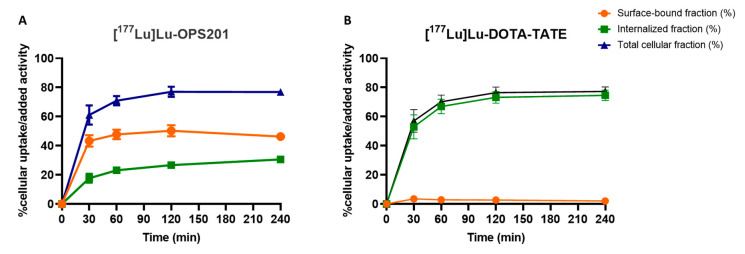
Cellular uptake of [^177^Lu]Lu-OPS201 (**A**) and [^177^Lu]Lu-DOTA-TATE (**B**). Data are representative of three independent experiments, each performed in triplicate.

[^177^Lu]Lu-OPS201 and [^177^Lu]Lu-DOTA-TATE experienced time-dependent uptake by plated HEK-SST_2_ cells at 37 °C (Figure 3). Both radioligands were taken up by about an equal amount at each individual time point. However, differences emerged when examining the cell surface bound and internalised fractions thereof. About half of the total added [^177^Lu]Lu-OPS201 was bound to the cell surface throughout the 4 h of incubation (i.e., 46 ± 2% at the end), but a substantial amount was also internalised slowly (i.e., 31 ± 1% at the end). By contrast, [^177^Lu]Lu-DOTA-TATE was almost entirely internalised (77 ± 3%), while only a minimal amount of radioligand remained surface-bound (2 ± 1%).

### 2.4. Redistribution of Cell Surface-Associated [^177^Lu]Lu-OPS201 at 37 °C

**Figure 4:**
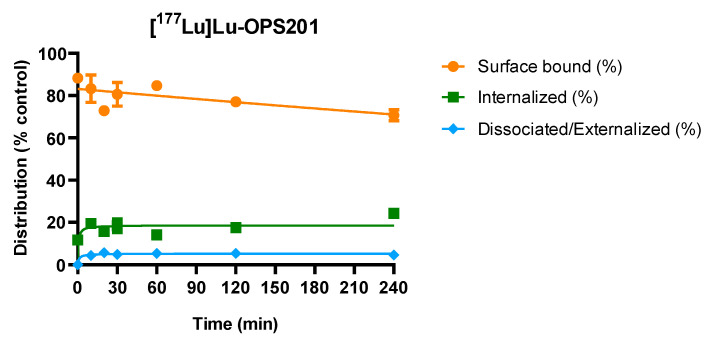
Redistribution of the cell surface-associated [^177^Lu]Lu-OPS201 at 37 °C, after allowing cell binding for 2 h at 4 °C. Each time point is the average of triplicate wells corrected for non-specific binding.

Cell surface-associated [^177^Lu]Lu-OPS201 was found to be internalised (Figure 4). After HEK-SST_2_ cells were pre-incubated with the radioligand for 2 h at 4 °C to impair radioligand internalisation, in accordance therewith, 54% of the added [^177^Lu]Lu-OPS201 remained bound on the cell surface, while only 7% was internalised. The new distribution of the radioligand, among the surface-bound, internalised and released fractions, remained constant after about 20 min under these experimental conditions. After 4 h of incubation at 37 °C, the initial surface-bound fraction was reduced by 17%, from 88% of the surface-bound activity at *t* = 0 to 71%. The internalised fraction on the other hand increased from 12% to 24%, and only <5% was released in the medium, suggesting that the newly internalised radioligands originated from the cell surface. Similar experiments with [^177^Lu]Lu-DOTA-TATE could not be performed due to its very low cell surface binding at 4 °C.

### 2.5. Release of Cell-Associated [^177^Lu]Lu-OPS201 with or without Unlabelled Competitors

**Figure 5:**
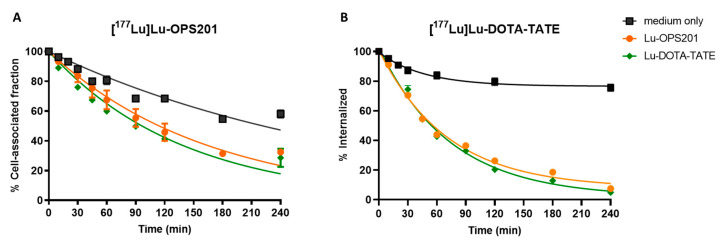
Dissociation/externalisation kinetics of [^177^Lu]Lu-OPS201 (**A**) at 37 °C, after allowing cell binding for 2 h at 4 °C. Data are plotted as percentage of the cell-associated fraction (surface-bound and internalised fraction at the onset of the washout step). Externalisation kinetics of [^177^Lu]Lu-DOTA-TATE (**B**) at 37 °C, after allowing its internalisation for 2 h at 37 °C. Data are plotted as percentage of the internalised fraction at the onset of the washout step.

The release of [^177^Lu]Lu-OPS201 by the intact cells at 37 °C is shown in Figure 5A. The medium was repeatedly refreshed in an attempt to limit any re-equilibration between free and cell-associated [^177^Lu]Lu-OPS201. This extra precaution may explain why appreciably more of the radioligand was released in naïve medium (Figure 5A): i.e., 40% versus <5% in Figure 4. In accordance with the rebinding paradigm, even more radioligand (i.e., 70% versus 40%) was released after 4 h when the “washout” medium contained an excess of either the unlabelled antagonist Lu-OPS201 or the agonist Lu-DOTA-TATE (Figure 5A). Based thereon, the actual, rebinding-free release of cell-associated [^177^Lu]Lu-OPS201 should take place with a t_1/2_ of about 130 min.

### 2.6. Externalisation of [^177^Lu]Lu-DOTA-TATE with or without Competitors

Internalised [^177^Lu]Lu-DOTA-TATE can be externalised, as shown by a similar experimental procedure as in Figure 5B, with the exception that the preincubation was also carried out at 37 °C to allow most of the cell-associated radioligand to be internalised and followed by a brief acid treatment to remove surface-bound agonist. The time-wise decrease thereof during the ensuing washout is displayed in Figure 5B. While this decrease was only partial after 4 h in naïve medium, it was nearly complete in the presence of an excess of Lu-DOTA-TATE and Lu-OPS201, yielding a difference compatible with the ability of the unlabelled competitors to effectively prevent the rebinding (and re-internalisation) of externalised radioligand molecules. Based thereon, the actual, rebinding-free release of cell-associated [^177^Lu]Lu-DOTA-TATE should take place with a t_1/2_ of about 55 min.

## 3. Discussion

The present in vitro study compared the SST_2_ binding characteristics and the cellular processing of the antagonist [^177^Lu]Lu-OPS201 and of the agonist [^177^Lu]Lu-DOTA-TATE on SST_2_-expressing HEK cells. Receptor binding and cellular processing studies provided distinct, albeit, complementary findings.

Saturation binding experiments on HEK-SST_2_ cell membranes showed that, despite a comparable high affinity, [^177^Lu]Lu-OPS201 was able to recognise about four times more specific binding sites than [^177^Lu]Lu-DOTA-TATE. This may explain, at least partially, the higher accumulation of [^177^Lu]Lu-OPS201 compared to [^177^Lu]Lu-DOTA-TATE in SST_2_-expressing tumours in both animal models [9,10] and patients [7]. The ability of [^177^Lu]Lu-OPS201 to recognise more binding sites compared with [^177^Lu]Lu-DOTA-TATE supports the use of radiolabelled SST_2_ antagonists not only in all NENs, including high grades, but also in tumours with lower SST_2_ density such as breast and small cell lung cancers [5], which is not the case for [^177^Lu]Lu-DOTA-TATE.

The saturation binding experiment results were corroborated by those of competition experiments. Indeed, while [^177^Lu]Lu-OPS201 dissociated completely and monophasically with time in the presence of an excess of its unlabelled counterpart and of the antagonist DOTA-BASS, the unlabelled agonists, Lu-DOTA-TATE and lanreotide, displaced no more than 30% of [^177^Lu]Lu-OPS201 binding. By contrast, [^177^Lu]Lu-DOTA-TATE dissociated entirely in the presence of both unlabelled antagonists as well as unlabelled agonists. Interestingly, [^177^Lu]Lu-DOTA-TATE dissociated completely and biphasically with time in the presence of an excess of its unlabelled counterpart or of the agonist lanreotide. This biphasic dissociation kinetic of [^177^Lu]Lu-DOTA-TATE may be attributed to its interaction as an agonist with G-protein coupled receptors (GPCRs) and non-coupled receptors, as reported for other agonistic ligands binding to GPCR [11,12].

Coupling of agonist–receptor complexes to G-proteins is well-known to confer high binding affinity [13,14]. Our findings point out that SST_2_ agonists can only bind with high affinity to part of the sites to which [^177^Lu]Lu-OPS201 is bound in HEK-SST_2_ cell membranes. This observation likely reflects the heterogeneous nature of agonist–receptor interactions with cell membrane preparations. In addition, while only a fraction of agonist–receptor complexes to G-proteins was able to bind with high affinity, the uncoupled complexes remained in low-affinity conformation [15,16,17,18]. Such segregation between coupled and uncoupled complexes may procure a net distinction between the number of antagonist and agonist high affinity sites in saturation binding experiments [19]. Our study may, hence, indicate high affinity binding of [^177^Lu]Lu-OPS201 to the SST_2_ targeted by the agonists and likely to additional sites of an unknown nature that are not labelled by the agonists. This deserves further investigations in complementary models to confirm this hypothesis of high clinical impact. Indeed, NENs are often treated with long-acting somatostatin analogues, such as lanreotide, which are commonly interrupted before the administration of radiolabelled somatostatin agonists, such as [^177^Lu]Lu-DOTA-TATE [2]. This practice is based on the assumption that the two somatostatin agonists compete for occupying the same somatostatin receptor sites. Our observations denote that the interruption of somatostatin agonists before treatment with radiolabelled analogues, which can worsen patient symptoms, may not be necessary when the radiolabelled somatostatin receptor is an antagonist.

Binding experiments on intact HEK-SST_2_ cells at 37 °C, which were used to compare the cellular distribution and potential rebinding of [^177^Lu]Lu-OPS201 and [^177^Lu]Lu-DOTA-TATE in the present study, offer the prospect to apprehend ligand–receptor interactions in a broader and also more physiologically relevant perspective [20]. Based on its ability to resist a brief mild acid treatment, more than 50% of [^177^Lu]Lu-DOTA-TATE was internalised after already 30 min of incubation. This finding concurs with previous studies with SST_2_ agonists [9,21,22] and with GPCR agonist-mediated desensitisation of cellular responses in general [23]. The agonist’s fast internalisation process may promote recycling of the ligand-bound GPCR, which in turn may explain the almost equal uptake of the agonist [^177^Lu]Lu-DOTA-TATE and the antagonist [^177^Lu]Lu-OPS201 by the cells, despite the recognition of a higher number of binding sites by the antagonist.

On the other hand, the interpretation of the cellular distribution of [^177^Lu]Lu-OPS201 is more challenging. Indeed, while the acid prompted the release of most of the cell surface-associated antagonist, a substantial fraction thereof was already refractory after 30 min of incubation at 37 °C. Moreover, when cells were pre-incubated with [^177^Lu]Lu-OPS201 at 4 °C, so that most of the binding resides at the surface, part of the acid-sensitive binding became internalised and remained resistant after a subsequent exposure to 37 °C. A similar redistribution has also been observed with other SST_2_ antagonists [24,25], as well as with gastrin-releasing peptide receptor antagonists [5]. It is of interest to note that other SST_2_ antagonists such as [^68^Ga]Ga-NODAGA-LM3 and DOTA-BASS do redistribute between acid-sensitive and acid-refractory fractions in the same way as [^177^Lu]Lu-OPS201, even though no internalisation thereof is detectable by immunofluorescence microscopy [24,26].

The possibility arises that all of the bound antagonist remains at the cell surface but part of the complexes acquires an acid-resistant conformation. Such induced-fit-like trans conformation is more likely to proceed when the membrane fluidity increases by raising the temperature [27], and the ratio between both conformations may also vary considerably from one antagonist to another [28]. This is in line with previous experiments on various antagonists concluding that the ratio between acid-sensitive and acid-refractory conformations may vary among different antagonists, although no internalisation is perceived [29,30,31]. Nonetheless, despite the widespread opinion that antagonists do not trigger the internalisation of their receptors [8,32], a number of GPCR antagonists have been reported to do so [33,34]. Such findings contributed to the understanding that GPCR agonists and antagonists are able to induce receptor internalisation through distinct mechanisms and that only the agonists trigger G-protein responsive receptor activation [35]. Pending further dedicated investigations, it remains uncertain whether the acid-refractory fraction of [^177^Lu]Lu-OPS201 is conformation- or location-related.

Both the release of [^177^Lu]Lu-OPS201 and [^177^Lu]Lu-DOTA-TATE by the intact cells at 37 °C was accelerated in the presence of a large excess of Lu-OPS201 and Lu-DOTA-TATE in the washout medium. Of note, both radioligands are hydrophilic and the accelerating effect of their unlabelled counterparts was slower for [^177^Lu]Lu-OPS201 than for [^177^Lu]Lu-DOTA-TATE (t_1/2_ of 130 versus 55 min, respectively). Compared to alternative interpretations such as allosteric interactions and intracellular events, these observations rather plead in favour of ligand rebinding [36,37]. To explain the accelerating effect of both competitors, this mechanism should imply that dissociated radioligand molecules are able to re-associate to unbound receptors that are present at the cell surface. A peculiar aspect of the procedure used in our study (i.e., Figure 5A,B) was that the 37 °C washout media were repetitively refreshed, aiming to at least reduce the accumulation, and, thus, the rebinding, of radioligand molecules when they were already dispersed in the bulk of the naïve medium. Such a “macroscopic” form of rebinding appeared to prevail for [^177^Lu]Lu-OPS201, as the amount of cell-associated [^177^Lu]Lu-OPS201 declined appreciably more in the refreshed naïve medium than in the undisturbed one (40% versus <5%, respectively after 4 h). On the other hand, [^177^Lu]Lu-DOTA-TATE declined at a slower rate in the refreshed naïve medium but faster in the presence of the unlabelled competitors, which seemingly confine the re-association or re-endocytosis of the [^177^Lu]Lu-DOTA-TATE via the receptors available of the cell surface. In line with this interpretation, Koenig et al. [38] reported that the peptidase-resistant SST_2_ receptor-bound agonist BM-23027 is able to repetitively cycle between the cell surface and intracellular compartments and also that, when freshly recycled, those molecules are able to re-activate the receptor in spite of their only limited accumulation in the extracellular medium. Together, the observed differences shed light on a firmer and potentially more localised form of rebinding [37,39].

It is noteworthy that the present study was conducted on recombinant cells and membranes thereof. G-protein coupled and non-coupled receptors may reside in distinct membrane microenvironments and their existence may vary in different cellular origins, e.g., recombinant and natural cells. Regardless, the slower release of [^177^Lu]Lu-OPS201 from HEK-SST_2_ cells in the present study is in line with its longer tumour retention, which led to higher tumour radiation doses in both preclinical in vivo models and human studies compared to [^177^Lu]Lu-DOTA-TATE [7,9,10].

## 4. Materials and Methods

All chemicals and solvents were obtained from Sigma-Aldrich (Merck KGaA, St. Louis, MI, USA), and used without additional purification. The peptide conjugates OPS201 and DOTA-TATE were kindly provided by OctreoPharm/Ipsen (Berlin, Germany). Non-carrier added ^177^Lu in aqueous 0.04 M HCl solution was obtained from ITM (Munich, Germany). All cell culture reagents were purchased from Bioconcept AG (Allschwil, Switzerland).

### 4.1. Preparation of Radioligands

[^177^Lu]Lu-OPS201 and [^177^Lu]Lu-DOTA-TATE were prepared by incubating 3–6 nmol of each conjugate with 30–150 MBq of [^177^Lu]LuCl_3_ at 95 °C for 30 min in ammonium acetate buffer (0.4 M, pH 5.1). The ratio of labelled-to-unlabelled conjugate ([^177^Lu]Lu-OPS201:OPS201 or [^177^Lu]Lu-DOTA-TATE:DOTA-TATE) was 1:6, based on the maximum specific activity of [^177^Lu]LuCl_3_. To obtain a structurally homogenous conjugate, an equivalent amount of unlabelled LuCl_3_ was added, and the radiolabelling solution was further incubated for 30 min at 95 °C. Quality control was performed by reverse phase high performance liquid chromatography (HPLC). The radiochemical yield (non-isolated, estimated by radio-HPLC) exceeded 95%, and the radiochemical purity exceeded 93% for all the preparations. The radioligands were diluted with 0.9% NaCl containing 0.05% human serum albumin to a concentration of 1 µM (stock solution).

### 4.2. Cell Culture, Intact Cells, and Cell Membranes

The HEK-293 cell line expressing the T7-epitope tagged human SST_2_ (HEK-SST_2_) was provided by Prof. Stefan Schulz (Institute of Pharmacology and Toxicology, Jena University Hospital, Jena, Germany). HEK-SST_2_ cells were cultured at 37 °C and 5% CO_2_ in DMEM containing 10% FBS, 100 U/mL penicillin, 100 µg/mL streptomycin, 200 µmol/mL L-glutamine, and 500 µg/mL geneticin (G-418).

To prepare cell membranes, HEK-SST_2_ cells were grown to confluence, mechanically disaggregated, washed with PBS (pH 7.4) and re-suspended in 20 mM of homogenisation Tris buffer (pH 7.5) containing 1.3 mM EDTA, 0.25 M sucrose, 0.7 mM bacitracin, 5 µM soybean trypsin inhibitor and 0.7 mM PMSF. The cells were homogenised using Ultra-Turrax, and the homogenised suspension was centrifuged at 500× *g* for 10 min at 4 °C. The supernatant was collected in centrifuge tubes (Beckman Coulter Inc., Brea, CA, USA). This procedure was then repeated 5 times. The collected supernatant was centrifuged in an ultra-centrifuge (Beckman) at 4 °C for 55 min at 20,000 rpm (approximately 49,000× *g*). Then, the pellet was re-suspended in 10 mM ice-cold HEPES buffer (pH 7.5), aliquoted, and stored at −80 °C. The protein concentration of those membrane suspensions was determined by the Bradford method, BSA as the standard.

In vitro assays on intact cells were performed with HEK-SST_2_ cells seeded in 6-well plates. The plates were pre-treated with a solution of 10% poly-lysine to promote cell attachment. HEK-SST_2_ cells (about 1 × 10^6^ cells) were incubated in 1% (*v*/*v*) FBS containing medium at 37 °C/5% CO_2_ overnight. The day after, the cells were washed with medium, followed by incubation with the adjusted medium volume for 1 h prior starting the experiment.

In all assays, non-specific binding of [^177^Lu]Lu-OPS201 was determined using 1000-fold excess of the unlabelled SST_2_ antagonist OPS202, also known as NODAGA-JR11, (where NODAGA is 1,4,7-triazacyclononane,1-glutaric acid-4,7-acetic acid). Non-specific binding of [^177^Lu]Lu-DOTA-TATE was determined using the same excess of the natural somatostatin-14 or DOTA-TATE. All reported values refer to specific binding (total minus non-specific binding). The radioactivity of all samples was measured in a gamma-counter (COBRA 5003, Packard Instruments). For all binding experiments with membranes, bound radioligand was separated from the one remaining in solution by rapid vacuum filtration, using an M-48 Brandel Cell Harvester (Alpha Biotech Ltd., Glasgow, UK). The filters were dried before being measured in the gamma-counter.

### 4.3. Association Kinetics and Related Saturation Binding on Cell Membranes

The association profiles of [^177^Lu]Lu-OPS201 and [^177^Lu]Lu-DOTA-TATE were studied at different concentrations, ranging from 0.05 to 1 nM, in HEK-SST_2_ cell membranes at 37 °C. Each assay tube contained 170 μL of binding buffer (20 mM HEPES, pH 7.4, containing 4 mM MgCl_2_, 0.2% BSA, 20 mg/L bacitracin, 20 mg/L PMSF and 200,000 KIU/L aprotinin). The incubation was initiated by adding 30 μL of radioligand solution at 10 times the final concentration and 100 μL of cell membrane suspension to yield 10 μg of protein per well. For the determination of the non-specific binding, 140 μL of the above binding buffer was added along with 30 μL of unlabelled ligand solution. For association kinetics, specific binding was plotted versus the incubation time, starting from 2 up to 60 min. For saturation specific binding, bound fractions were plotted versus the corresponding radioligand concentration at equilibrium. However, given that total binding occasionally exceeds 10% of the total amount of added radioligand, radioligand concentrations were corrected for the ligand depletion effect by the “One site Total, accounting for ligand depletion” equation (GraphPad Software Inc., Prism 7, San Diego, CA, USA). The dissociation constant (K_D_) and maximal binding capacity (B_max_) values were calculated using GraphPad.

### 4.4. Dissociation Kinetics on Cell Membranes

HEK-SST_2_ cell membranes (10 µg/well) were pre-incubated with 0.1 nM [^177^Lu]Lu-OPS201 or 0.15 nM [^177^Lu]Lu-DOTA-TATE in binding buffer for 1 h to reach equilibrium (*t* = 0). The dissociation of those radioligands was initiated by adding a 1000-fold excess of either unlabelled Lu-OPS201, Lu-DOTA-TATE, DOTA-BASS or lanreotide and monitored for up to 3 h. To monitor the stability of the radioligand–receptor complex during this extended time period, the membranes were incubated with radioligand for up to 4 h.

### 4.5. Radioligand Binding to and Internalisation by Intact Cells

The internalisation rates of [^177^Lu]Lu-OPS201 and [^177^Lu]Lu-DOTA-TATE were studied in HEK-SST_2_ cells seeded in 6-well plates (10^6^ cells/well) and incubated at 37 °C with medium containing [^177^Lu]Lu-OPS201 or [^177^Lu]Lu-DOTA-TATE (2.5 nM) either alone or in the presence of blocking ligand to distinguish between specific and non-specific binding and uptake. After 0.5, 1, 2 and 4 h (one plate per time point), the medium was removed, and the plated cells were quickly washed twice with ice-cold PBS. The cells were then treated twice for 5 min with ice-cold glycine solution (0.05 M, pH 2.8) to detach the cell surface-bound radioligand (acid releasable) [40]. Afterwards, the cells containing the internalised radioligand were detached with 1 M NaOH at 37 °C and collected for measurement. The amount of specific cell surface-bound and internalised radioligand is expressed as a percentage of the total applied activity after subtracting the non-specific values.

### 4.6. Sub-Cellular Redistribution of Cell Surface-Bound [^177^Lu]Lu-OPS201 at 37 °C

The cell surface-bound internalisation and dissociation rates of [^177^Lu]Lu-OPS201 were studied in HEK-SST_2_ cells seeded in 6-well plates (10^6^ cells/well), which were first placed on ice for 30 min. The experiment was started by adding 2.5 nM [^177^Lu]Lu-OPS201 to the medium, and the incubation was carried out at 4 °C for 2 h to prevent internalisation of the radioligand. Afterwards, the cells were washed with ice-cold PBS, and 1 mL/well of fresh pre-warmed (37 °C) medium was added. One plate was immediately treated with ice-cold glycine solution, and then the cells were detached with NaOH to determine the initial surface-bound and internalised fraction. The other plates were incubated at 37 °C for 10, 20, 30, 60, 120 or 240 min (one plate per time point) before the same treatment. At the specified time points, the amount of radioactivity, presented as cell surface-bound, internalised and in medium redistributed radioligand, was expressed in percentage of the cell-associated fraction (surface bound and internalised uptake) at the onset of the redistribution step as control. All reported values have been corrected for non-specific uptake.

### 4.7. Dissociation of Cell Surface-Bound [^177^Lu]Lu-OPS201 by Intact Cells

The dissociation rate of the antagonist [^177^Lu]Lu-OPS201 was studied in plated HEK-SST_2_ cells after incubation of [^177^Lu]Lu-OPS201 for 2 h at 4 °C and washed with ice-cold PBS. The cells were then incubated at 37 °C under three different conditions: (a) in presence of fresh medium with 1% FBS alone, (b) in the presence of a 1000-fold excess of Lu-OPS201 and (c) in the presence of a 1000-fold excess of Lu-DOTA-TATE. At preselected time points, the medium was removed for quantification and replaced by fresh pre-warmed (37 °C) medium (either alone or with the same unlabelled competitor). The use of unlabelled competitors has been shown to prevent the rebinding of freshly dissociated radioligands to their receptors in plated cells [37]. At the end, the cells were treated with NaOH and collected for measurement. All values were also normalised as described above.

### 4.8. Externalisation of [^177^Lu]Lu-DOTA-TATE by Intact Cells

Plated HEK-SST_2_ cells were pre-incubated with 2.5 nM [^177^Lu]Lu-DOTA-TATE for 2 h at 37 °C to allow its internalisation. The medium was then removed, and the cells were treated twice for 5 min with ice-cold glycine solution (0.05 M, pH 2.8) to remove the surface-bound agonist radioligand. The cells were afterwards incubated at 37 °C under the same three different conditions as above. At preselected time points, ranging from 10 min up to 4 h, the medium was removed for quantification and replaced by fresh pre-warmed (37 °C) medium (alone or with the same unlabelled competitors). At the end, the cells were treated with NaOH and collected for measurement. All reported values were also normalised as described above.

In both aforementioned experiments, the 37 °C washout medium was repeatedly refreshed in an attempt to limit any re-equilibration between free and cell-associated radioligand and to reduce the accumulation (and thus the rebinding) of radioligand molecules when they were already dispersed in the bulk of the naïve medium. All data were fitted according to the one-phase exponential decay equation (GraphPad Software Inc., Prism 7).

### 4.9. Data and Statistical Analysis

Data are presented as mean ± standard deviation. All statistical procedures were performed using GraphPad Prism, version 7.0 (RRID: SCR_002798; GraphPad Software Inc.; San Diego, CA, USA).

## 5. Conclusions

The present in vitro study found that despite a comparable high affinity, [^177^Lu]Lu-OPS201 recognised at least four times more receptor binding sites than [^177^Lu]Lu-DOTA-TATE. Moreover, [^177^Lu]Lu-OPS201 showed faster association, slower dissociation and longer cellular retention than [^177^Lu]Lu-DOTA-TATE. These properties can lead to a therapeutic efficacy gain with the antagonist [^177^Lu]Lu-OPS201 compared to the agonist [^177^Lu]Lu-DOTA-TATE, regardless of the localisation at the sub-cellular level. Furthermore, the study indicates that interruption of somatostatin agonists before treatment with radiolabelled analogues may not be necessary if SST_2_ antagonists are used. Finally, the presented findings on intact SST_2_-expressing cells and membranes may provide useful hints for further research on more physiologically relevant material.

## Figures and Tables

**Table 1:** Summary of the dissociation rate constant (k_off_ in min^−1^) determined for both radioligands, [^177^Lu]Lu-OPS201 and [^177^Lu]Lu-DOTA-TATE, in the different experimental settings.

Competitor	[^177^Lu]Lu-OPS201	[^177^Lu]Lu-DOTA-TATE
	k_off_ (min^−1^) in HEK-SST_2_ Membranes	
Lu-OPS201	0.017 ± 0.002	0.004 ± 0.0002
Lu-DOTA-TATE	n.a.	0.019 ± 0.005 (k_2_) 0.38 ± 0.05 (k_1_)
DOTA-BASS	0.018 ± 0.002	0.005 ± 0.0003
Lanreotide	n.a.	0.012 ± 0.002 (k_2_) 0.32 ± 0.03 (k_1_)

n.a. not applicable.

## Data Availability

The data supporting the findings of this study are available from the corresponding author upon reasonable request.
